# In Vitro Probiotic Potential and Safety Evaluation (Hemolytic, Cytotoxic Activity) of *Bifidobacterium* Strains Isolated from Raw Camel Milk

**DOI:** 10.3390/microorganisms8030354

**Published:** 2020-03-02

**Authors:** Iqra Yasmin, Muhammad Saeed, Wahab Ali Khan, Adnan Khaliq, Muhammad Farhan Jahangir Chughtai, Rabia Iqbal, Saima Tehseen, Saima Naz, Atif Liaqat, Tariq Mehmood, Samreen Ahsan, Saira Tanweer

**Affiliations:** 1 Department of Food Science and Technology, Government College Women University, Faisalabad 38040, Pakistan; rabi1300@hotmail.com (R.I.); dr.saimatehseen@gcwuf.edu.pk (S.T.); 2 Barani Agricultural Research Institute, Chakwal 48800, Pakistan; 3 National Institute of Food Science and Technology, Faculty of Food, Nutrition and Home Sciences, University of Agriculture, Faisalabad 38040, Pakistan; 4 Department of Food Science and Technology, Khwaja Fareed University of Engineering and Information Technology, Rahem Yar Khan 64200, Pakistan; adnan1103.ft@gmail.com (A.K.); m.farhan.chughtai@hotmail.com (M.F.J.C.); atifliaqat09@gmail.com (A.L.); tariq.mehmood@kfueit.edu.pk (T.M.); samreenahsan.ft@gmail.com (S.A.); 5 Department of Clinical Nutrition, Nur International University, Lahore 54000, Pakistan; saimanaz2109@gmail.com; 6 University College of Agriculture and Environmental Sciences, Islamia University, Bahawalpur 63100, Pakistan; sairatanweer1116@gmail.com

**Keywords:** probiotic potential, antimicrobial activity, camel milk: B-11, pathogens

## Abstract

The present study was designed to isolate *Bifidobacterium* strains from raw camel milk and to investigate their probiotic characteristics. Among 35 isolates, 8 were identified as Gram-positive, catalase negative, non-spore forming, non-motile and V or Y shaped rods. B-2, B-5, B-11, B-19 and B-28 exhibited good survival at low pH and high bile salt concentration. Most of the isolates were resistant to nalidixic acid, fusidic acid, polymyxin B, neomycin, streptomycin, gentamicin, rifampicin and kanamycin. Furthermore, the production of exopolysaccharides (EPS), adhesion characteristics, antioxidant properties, antagonistic activities, nitrite reduction and cholesterol assimilation were also studied. Isolate B-11 was chosen because it exhibited most of the probiotic properties among all the tested isolates. It is identified as the member of *Bifidobacterium longum* group through 16S rRNA gene sequencing and named as *B. longum* B-11. *B. longum* B-11 was further selected for in vivo attachment to rat intestine and scanning electron micrographs revealed that attachment of a large number of rods shaped bacterial cell. Our findings suggest that *B. longum* B-11 processes excellent attributes to be used as potential probiotic in the development of functional probiotic food.

## 1. Introduction

Probiotics are the live microorganisms that confer certain health benefits to the host beyond basic nutrition (FAO/WHO 2006). They are proposed to provide certain health benefits i.e., balance gut microflora, alleviate gastrointestinal infections, inhibit the growth of pathogenic bacteria, strengthening the barrier function of the gut, improve immunity, assimilation of serum cholesterol, prevention from irritable bowel syndrome, reduce hypertension, prevent diarrhea and etc. [1]. Different genera of *Lactococcus*, *Lactobacillus*, *Streptococcus*, *Bifidobacterium* and various yeast strains have been well-studied probiotics. The main attribute in the identification and application of probiotics in the food matrix is they should be Generally Recognized As Safe (GRAS) for human consumption and provide probiotics microorganisms in sufficient amount at the time of consumption [2]. Selection of probiotics strain is also important for application in the food industry, their ability to survive and to keep their functional properties intact during processing and storage under harsh condition i.e., spray drying and freezing, along their survival during gastric transit [3].

Bifidobacteria are catalase-negative, Gram-positive, non-motile, non-spore forming, anaerobes belonging to phylum *Actinobacteria*. Bifidobacteria are saccharolytic organisms that hydrolyzed or metabolized carbohydrates. Bifidobacteria appear in V or Y shape, short rods, uniform, single or branched, bifurcated or clubbed [4]. The isolation of bifidobacteria as a potential probiotic culture, the antimicrobial agent is promising in a wide range of biotechnological and biomedical applications. These probiotics potentially used as microbial culture in food systems due to its safety aspects and various functional attributes [5]. 

Bifidobacteria exhibited tremendous biotechnological and functional attributes including the ability to survive in harsh gastrointestinal conditions that seem to vary among different strains of *Bifidobacterium* due to phenotypic and genotypic variations within the species. FAO and WHO have developed basic criteria for the screening and selection of probiotics. A probiotic strain must survive and colonized in the host’s gastrointestinal tract. Probiotics ought to possess cell surface properties i.e., auto-aggregation, cell surface hydrophobicity, should tolerate low pH of stomach and bile salt [6,7,8,9]. Additionally, probiotics must have functional properties i.e., cholesterol assimilation, antagonistic and antioxidant activities to exert positive health benefits to the host [10]. Besides the functional attributes, the safety evaluation of probiotic such as resistance to antibiotics, blood hemolytic activity, antimutagenic activity, cytotoxicity, intestinal inflammatory cytokines, and the histopathological test must be performed before its contact with an animal or human. 

Camel milk is gaining consumer’s interest due to its various nutritional and therapeutic attributes. In hot, arid and semi-arid areas of Asia and Africa, camel milk is used as raw or in fermented form. In these regions, camel milk is considered a major dairy source for human nutrition. In the Middle East and Africa, mostly fermented camel milk products are being consumed. Studied revealed that camel milk is a rich source of beneficial microflora especially Lactic Acid Bacteria (LAB) and Bifidobacteria. Saudi Arabia, Somalia, and the United Arab Emirates (UAE) are the top camel milk producing countries all around the world. LAB is successfully isolated from camel milk and fermented camel milk products [11,12,13,14,15,16,17,18,19,20]. To the best of my knowledge, to date, no work has been published regarding the isolation of bifidobacteria from camel milk. 

In previous years, the use of additive with antimicrobial properties gaining attention to introduce new and natural protective agents that don’t induce antimicrobial-resistance genes. This bioprotective agent can be effectively used as an alternative of antibiotics for food preservation, food safety and biomedical application. Among natural preservatives, probiotics are one of the live alternatives due to the synthesis of organic acid and bacteriocins [21]. Recent studies on the antimicrobial activities of probiotics are prime importance in the control of pathogenic and food spoilage microorganisms. The effectiveness of bifidobacteria to control pathogenic bacteria has been well studied. Probiotics also have been studied for their effectiveness on the reduction of blood serum cholesterol. Studies revealed that consumption of probiotic dairy products significantly reduces total serum cholesterol and low-density lipoprotein (bad cholesterol) [22]. The probiotic potential and preservative effect of microorganisms depend upon isolation sources and species. It is a complex task to select the origin of probiotics for isolation but most of the probiotics are isolated from human origin and fermented products. Recently, more attention has been paid to isolate probiotic from indigenous sources to introduce new resources to improve the host’s health. The gut microbiome composition differs from person to person due to diversity in geographic expanse, foods, habitat and lifestyle [23]. Thus, it is interesting to design cost-effective probiotic starter culture which has equal opportunity to be used as a probiotic and a food starter culture. 

The aim of the current study was to isolate, characterized bifidobacteria from raw camel milk and to provide more inclusive report for their probiotic potential. The presumptive isolates were assessed for tolerance to gastric juice and bile salt. Additionally, probiotic potential and safety evaluation was confirmed through antioxidant, antibacterial activity, exopolysaccharide production, cholesterol assimilation, antibiotics susceptibility, hemolytic activity and cytotoxicity. On the basis of preliminary analysis, one of the best strains was identified through 16 rDNA sequencing.

## 2. Materials and Methods 

### 2.1. Chemicals 

All media, chemicals, reagent and kits used in this study were of analytical grade and purchased from St. Louis, MO, USA., Sigma-Aldrich Chemicals Ltd., Merck and Co. Inc. and White House Station, NJ, USA.

### 2.2. Sample Collection and Isolation

Forty-five raw camel milk samples were collected from local lactating healthy camels (*Camelus dromedaries*) from different regions of Punjab, Pakistan. The samples were collected into a sterilized container. Due to long-distance, samples were placed in iceboxes until delivered to the Food Microbiology and Biotechnology Laboratory, NIFSAT, UAF and immediately analyzed upon arrival. Samples were homogenized (10% w/v) in sterile phosphate buffer using stomacher. After preparation of dilutions in sterile saline (0.85% *w/v* NaCl), the samples were plated on de Man Rogosa Sharpe (MRS) agar (Difco, Sparks, MD, USA) supplemented with L-cysteine 0.05% (w/v) (Sigma Aldrich, St. Louis, MO, USA). Plates were incubated anaerobically in an anaerobic chamber (Bactron Shel Lab II-1, Sheldon Manufacturing Inc., Cornelius, OR, USA) at 37 °C for 48 h. After incubation, morphologically dissimilar colonies were selected and re-streaked on an agar plate to get pure isolates. 

### 2.3. Preliminary Identification and Screening of Bifidobacterium 

All isolates (35) were analyzed on the basis of morphology, Gram staining, catalase test, motility test and endospore test. After preliminary screening, 8 isolates were identified as a member of genus *Bifidobacterium* on the basis of Gram-positive, catalase-negative, non-spore forming, non-motile and V or Y shaped rods. These isolates were further analyzed for growth characteristics i.e., growth at various temperatures (10 °C, 37 °C and 45 °C), growth at different NaCl concentrations (3%, 5% and 7%) and growth at various pH (4.4 to 9.6). These isolates were also analyzed for their ability to produce CO_2_ and ferment various sugar by using API 50CH kit. Microscopic examination was performed by using the EVOS FL Auto Cell Imaging System (Thermo Fisher Scientific, Waltham, MA, USA). The pure isolate was preserved in MRS broth in 30% glycerol at –80 °C as a frozen stock. The culture was reactivated prior to use by sub-culturing at least 3 times in MRS broth. 

### 2.4. In Vitro Screening of Probiotic Potential of Isolates

#### 2.4.1. Survival under Gastrointestinal Conditions and Phenol Tolerance

Isolates were screened for their ability to withstand in harsh gastrointestinal conditions i.e., low gastric pH, bile salt and 0.4% (w/v) phenol. Isolates were cultivated in MRS broth to get the desired cell number (10^9^ logs CFU/mL) after 18 h of incubation at 37 °C. The simulated gastric juice (SGJ) containing pepsin (3 g/L) was prepared in sterile saline (0.85%, *w/v* NaCl) and adjusted the pH 2, 3 by using 1 M HCl. 1 mL (10^9^ logs CFU/mL) of overnight grown cell suspension was mixed with 9 mL of sterilized SGJ under aseptic conditions and incubated at 37 °C for 2 h. Similarly, tolerance to bile salt and phenol was evaluated by inoculating bacterial suspension (1 mL containing 10^9^ logs CFU/mL) in MRS broth tubes containing a various concentration of bile salt (0.1%, 0.5% and 1% w/v) and phenol (0.4%, w/v) [24]. After incubation, the viable count was determined by plating and expressed as CFU/mL [25].

#### 2.4.2. Production of Exopolysaccharides (EPS)

The EPS production was determined as described by Liu, et al. [26]. 1% (v/v) of each isolated bacterial culture (10^9^ CFU/mL) was inoculated into MRS broth supplemented with 2% (w/v) glucose and incubated (37 °C for 24 h). After completion of incubation, cell pellets were removed by centrifugation (4000× g, at 4 °C for 10 min). Trichloroacetic acid 4% (w/v) was added and mixture was vortex at 4 °C for 3 h. Precipitated proteins were removed through centrifugation (22,000× g, 20 min), supernatant was concentrated through evaporation. Four volumes of ethanol (95%, v/v) was added to precipitated EPS, the mixture was centrifuged (22,000× g, at 4 °C for 20 min) and stored for 24 h at 4 °C. EPS was dialyzed (6000 Da to 8000 Da molecular weight) for 48 h at 4 °C and freeze-dried to make powder. The EPS amount was calculated by following the phenol sulfuric acid method [27].

#### 2.4.3. Auto-Aggregation Assay

The auto-aggregation assay was accomplished by following the method as described by Xu, et al. [28] with little modifications. Overnight culture of all the tested isolates were collected after centrifugation (4000× g, at 4 °C for 10 min). The cells were washed two times with phosphate buffer saline (PBS) and re-suspended in PBS to reach an absorbance of (0.5 ± 0.02) at 600 nm at 0 h (A_0_). Then, 1 mL of each bacterial suspension was vortexed for 5 sec and incubated (37 °C for 2 h, 6 h, 12 h and 24 h). Supernatant absorbance was measured at 600 nm after 2 h, 6 h, 12 h and 24 h of incubation (A_2_) of incubation (Equation (1)).

(1) Autoaggregation (%)= 1 − (A2/A0)×100

Whereas, A_0_ = Initial absorbance at 0 h; A_2_ = Final absorbance after 2 h, 6 h, 12 h and 24 h of incubation

#### 2.4.4. Cell Surface Hydrophobicity

Cell surface hydrophobicity was evaluated via all the 8 isolated bacteria that have the ability to bind with hydrocarbons by following the method as described by Kotzamanidis, et al. [29] with slight modifications. Briefly, overnight grown cells were collected after centrifugation (4000× g, at 4 °C for 10 min). The cells were washed two times with PBS having pH 7 and resuspended in PBS to reach an absorbance of (0.5 ± 0.02) at 600 nm at 0 h (A_0_). After that, 1 mL of hydrocarbon (xylene) was mixed separately with 3 mL cells suspension and pre-incubated at 37 °C for 10 min. The cell suspension and hydrocarbon mixture were vortexed for 2 min and kept for 20 min for phase separation (water and hydrocarbon phase). After the collection of the aqueous phase, absorbance was measured at 600 nm (A_1_) (Equation (2)).

(2) Hydrophobicity (%) = (1 − A1/A0) × 100

Whereas, A_0_ = Initial absorbance, A_1_ = Final absorbance 

#### 2.4.5. DPPH Free Radical Scavenging Activity 

DPPH free radical activity of isolates was analyzed by following the method described by Chen, et al. [30]. Briefly, 100 µL of freshly prepared cells (10^9^ CFU/mL) was mixed with 1 mL of (0.05 mM) DPPH solution. The mixture was mixed and kept for 30 min in a dark place. The absorbance was recorded at 517 nm and DPPH scavenging activity was calculated as given in (Equation (3)).

(3) DPPH scavenging activity (%)= 1− (A sample − A blank/A control) × 100

Whereas A _sample_ = Bacterial cell and DPPH solution; A _blank_ = A mixture of methanol and bacterial cells; A _control_ = DPPH solution 

#### 2.4.6. Resistance to Hydrogen Peroxide

The resistance of isolates to hydrogen peroxide was determined by a modified method described by Oberg, et al. [31]. 10 mL of freshly prepared culture were mixed with 10 mL 0.85% (w/v) NaCl in different test tubes with varying concentrations of H_2_O_2_ (0.5, 1.0 and 1.5 mM). The viability of isolates was determined by withdrawing samples after 60 min of incubation.

#### 2.4.7. Depletion of Sodium Nitrite

The depletion of sodium nitrite by isolates was documented by following the method as described by Wu, et al. [32] with minor changes. 1 mL (1500 µg/mL) of sterilized sodium nitrite solution was mixed with 9 mL of MRS broth (pH 6.5) to making it 150 µg/mL. 100µL freshly prepared bacterial culture (10^9^ CFU/mL) was inoculated and incubated anaerobically for 48 h at 37 °C. In the case of control samples, sterile water was used instead of inoculum. The colorimetric nitrite method was used to measure initial and final absorbance at 538 nm by following the method described by Yan, et al. [33]. 5 mL of a mixture containing (inoculum, MRS broth, nitrite) was deproteinated and defatted by using 10 mL of ZnSO_4_ (0.42 mol/L), then the mixture was filtered. 1 mL of each these three-color developing solutions (0.2% (w/v) sulfanilamide, 0.1% (w/v) N-1-naphtyethylene diamine dihydrochloride and 44.5% (v/v) HCl) were added in filtrate and mixed it well. The mixture was kept in dark place at 37 °C for 5 min. The optical density of the color mixture was recorded at 538 nm against a blank. 3000 µg/mL standard sodium nitrite solution was prepared and a standard curve was constructed by following the same protocol (Equation (4)).

(4) Nitrite depletion (%) = (1 − Ci/Cf) × 100

Whereas, C_i_ = Amount of nitrite present in MRS broth at 0 h; C_f_ = Amount of nitrite present in MRS broth after 48 h.

#### 2.4.8. Antibacterial Activity

Antibacterial activity of isolates against three human pathogens i.e., *Escherichia coli* ATCC 25922, *Salmonella typhimurium* ATCC 14,028 and *Staphylococcus aureus* ATCC 25,923 were determined through ager well diffusion method [10]. 1 mL of the freshly prepared cells of each isolate was filtered by using a 0.2 µm syringe filter. Each indicator pathogen was overlaid on Muller Hinton agar plates. 50 µL of culture filtrate (cell-free supernatant) was poured into 7 mm diameter wells created with borer. The plates were incubated at 37 °C for 24 h. The clear zone diameter of each isolate against each indicator pathogen was measured after incubation. The diameter of clear zone was described as: no inhibition (0 mm), diameter between 0–3 mm (weak inhibition), diameter between 3–6 mm (good inhibition) and diameter greater than 6 mm (strong inhibition) [10]. 

#### 2.4.9. Cholesterol Reduction Assay

Cholesterol stock solution was prepared by dissolving 30 mg of water-soluble cholesterol (polyoxyethanyl-cholesterol sebacate) in 10 mL distilled water and filtered (0.45 µm). MRS broth containing 100 µL/mL cholesterol stock and 0.30 g/100 mL bile salt (oxgall) was inoculated with 1% of freshly prepared culture and incubated (37 °C for 24 h). The samples were withdrawn after a predetermined time interval (6, 12, and 24 h) and centrifuged (4000× *g*, at 4 °C for 10 min). The supernatant was used to determine cholesterol content by using modified colorimetric method Rudel and Morris [34] with slight modification [35]. 2 mL of this supernatant was mixed with 2 mL (33% w/v) KOH and 2 mL ethanol (96%). This mixture was vortexed for 2 min, incubated at 37 °C for 30 min and cooled to room temperature. After co40oling, 3 mL of hexane and 2 mL of Milli-Q water was added and vortexed for 2 min. The mixture was kept for separation of two phases, hexane layer was separated (upper layer) and evaporated. 2 mL of o-phthalaldehyde reagent (50 mg OPA dissolved in 100 mL glacial acetic acid) was added and vortexed for 2 min. 0.5 mL H_2_SO_4_ (98% purity) was added into this mixture and vortexed. After 10 min rest at room temperature, absorbance was recorded at 550 nm. Cholesterol removal (%) was calculated as given in Equation (5).

(5) $$\mathrm{Cholesterol}\;\mathrm{removed}\;\left(\%\right)=\left\{\frac{100\;-\;\mathrm{residual}\;\mathrm{cholesterol}\;\mathrm{after}\;24\;h\;\mathrm{of}\;\mathrm{incubation}}{100}\right\}\times 100$$

### 2.5. Safety Evaluation of Isolates 

#### 2.5.1. Antibiotic Susceptibility

The antibiotic susceptibility of isolates was tested against selected antibiotics (ampicillin, cephalothin, chloramphenicol, cloxacillin, erythromycin, nalidixic acid, fusidic acid, polymyxin B, neomycin, streptomycin, gentamicin, rifampicin, kanamycin, novobiocin, penicillin, spectinomycin, tetracycline and vancomycin) by using antibiotic disc diffusion method on MRS agar plates as described by Vijayakumar, et al. [36]. MRS agar plates were prepared by swabbing the overnight culture of tested isolates. Antibiotic discs were placed on solidified MRS agar and give them 30 min for antibiotic diffusion and after that incubated (37 °C for 48 h). The zone of inhibition was measured for each antibiotic disc after the completion of incubation. The antibiotic susceptibility was differentiated by measuring zone of inhibition i.e., <8 mm resistant, 8–10 mm moderate and >10 mm susceptibility.

#### 2.5.2. Hemolytic Activity

The hemolytic activity of isolates was determined by using Columbia agar containing 5% (w/v) sheep blood and the plates were incubated at 37 °C for 48 h. After incubation, the hemolytic activity of isolated strains was evaluated and classified on the basis of lysis of red blood cells in the medium around the colonies. The green zones around colonies (α-hemolysis), clear zones around colonies (β-hemolysis) and no zones around colonies (γ-hemolysis) on Columbia blood agar plates. Only strains with γ-hemolysis are considered as safe [37]. 

#### 2.5.3. Cytotoxicity 

In vitro cytotoxicity activity isolates were determined according to the procedure reported in Mohanty, et al. [38] with slight modification. The cytotoxic (anti-cancer) activity of isolated strains was carried out using 3-(4,5-dimethyl-thiazol-2-yl)-2,5-diphenyltetrazolium bromide (MTT) assay. This assay measured the development of blue formazan product as an MTT reduction by mitochondrial dehydrogenase, which is the indicator of cell viability and normal functioning of mitochondria. Briefly, exponentially growing 3 × 10^4^ Caco-2 cells/well (100 μL/well) were incubated in 96-well plates and cultured for 24 h. The culture medium was removed after 24 h and each tested isolate (10^7^ CFU/mL) cell-free filtrate was added to the culture medium. The Caco-2 cells were further incubated for 8 h and 24 h. After incubation, the percentage of cells surviving was counted using MTT assay. MTT solution (100 μL/mL) was added to each well. After 2 h of incubation at 37 °C, 100 μL of dimethylsulfoxide (DMSO) was added to dissolve the blue crystals and absorbance was read. The optical density was measured at 570 nm using a microplate reader. 

### 2.6. Molecular Identification of Isolate (B-11)

The selected potential probiotic *Bifidobacterium* isolate (B-11), that possessed most of the probiotics functional characteristics were identified on the basis of 16S rRNA gene sequencing. Bacterial genomic DNA was extracted by following the phenol-chloroform extraction method as described by Martinez, et al. [39] and PCR was performed by using universal 16S primers (8F and 1391R). Amplicons were electrophoresed using 1% agarose gel and purified by QIAquick PCR purification kit (Qiagen, USA) and quantified using Nano Drop (ND-1000) Spectrophotometer. The gene sequencing was done by the Genomics Core Facility at Michigan State University, USA. The obtained sequence was compared and molecularly identified with the available nucleotide database from the NCBI GenBank using the BLAST search (https://blast.ncbi.nlm.nih.gov/Blast.cgi). After gene sequencing, the pure isolate was labeled as *Bifidobacterium longum* B-11. The obtained sequence was deposited into NCBI GenBank and allotted NCBI accession number (MH041649.1). 

### 2.7. Attachment of B. Longum B-11 to Rats Intestine; In Vivo Studies

*In vivo* studies were carried out on 10 Sprague Dawley rats (age: 3–4 weeks; weighing 15–22 g). The rats were accommodated in the animal room of the National Institute of Food Science and Technology (NIFSAT), University of Agriculture, Faisalabad after obtaining ethical approval granted from the head of NIFSAT, University of Agriculture, Faisalabad Pakistan. During the entire period, the environment in the animal room was maintained (temperature 23 ± 2 °C; relative humidity 55 ± 5%) with a 12 h light/dark cycle. The animals were fed on normal diet and tap water ad libitum for one week before the start of the trail for adaptation and to confirm their normal growth behavior. Before the start of the experiment, animal feed and water intake were observed to be free from any contamination and pathogenic infection. Rats were randomly divided into two groups (G_0_ and G_1_), having 5 rats in each group. G_0_ group were administrated with normal diet while G_1_ were administrated *B. longum* B-11 (10^7^–10^9^ CFU/mL) as a single dose along with normal diet for a period of one week. Administration was achieved through intragastric gavage. 

### 2.8. Scanning Electron Microscopy of Rats Intestine

After one week of oral administration of *B. longum* B-11, the Sprague Dawley rats were sacrificed by cervical dislocation and their intestines were removed gently. The segmented intestine was opened and washed with PBS and fixed in 4% glutaraldehyde for 1 h [40]. The dehydrated samples were placed on aluminum stubs and coated with gold-palladium [41]. Field emission scanning electron microscope (FE-SEM) (S4700, Hitachi High-Technologies Corporation, Japan) was used to observe the attachment of *B. longum* B-11 on the intestinal surface and micrographs were taken with the same microscope.

### 2.9. Statistical Analysis

All the experimental trials were carried out in duplicate and for each trial, analysis was performed in triplicate. The results were presented as mean ± standard deviation (SD). The data collected in this study were analyzed statistically through a completely randomized design (CRD) using Statistix 8.1. Level of significance (*p* < 0.05) was estimated by using the analysis of variance technique (ANOVA) with two factor factorials under CRD followed by Tukey’s multiple comparison test. 

### 2.10. Ethical Approval

All procedures performed in studies involving animals were in accordance with the ethical standards of the institution or practice at which the studies were conducted.

## 3. Results 

### 3.1. Isolation and Identification of Presumptive Bifidobacterium Isolates

A total of 35 isolates (*Bifidobacterium* and LAB) from raw camel milk were preliminary identified. Out of these, eight isolates (B-2, B-5, B-11, B-14, B-19, B-21, B-28 and B-33) showed an appearance of bifidobacteria on the MRS medium and selected for a further experiment on the basis of cultural, morphological, microscopic, physiological and biochemical characteristics. Most of the isolates formed white to off white, shiny round colonies on an agar plate. Preliminary characterization suggested that selected isolates were all Gram-positive, rods (V or Y) shaped, catalase-negative, non-motile and non-spore forming (Table 1).

### 3.2. In Vitro Screening of Probiotic Potential of Isolates

#### 3.2.1. Survival under Gastrointestinal Conditions and Phenol Tolerance

**Figure 1:**
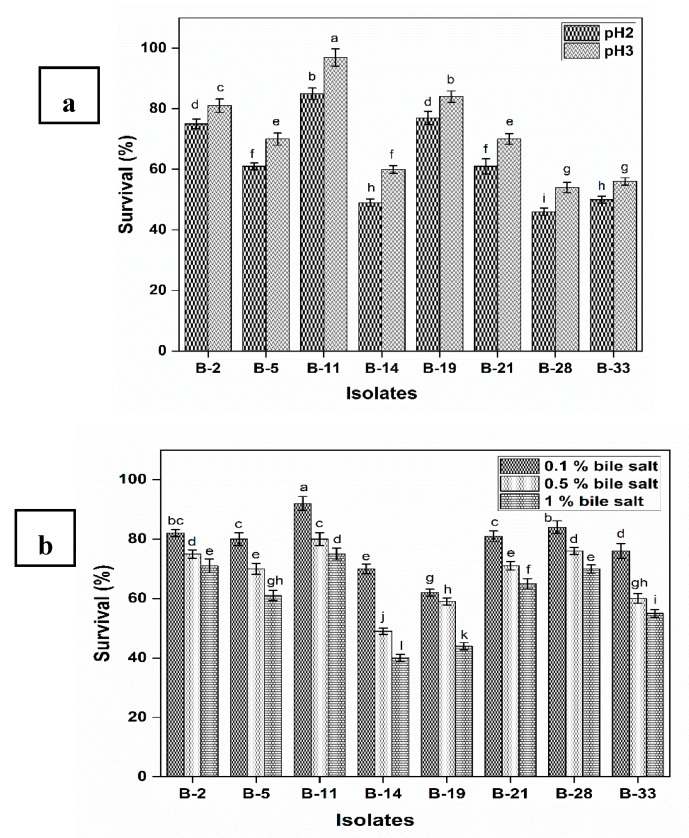
Survival (%) of isolates under gastrointestinal conditions (**a**) Tolerance to low pH (2 and 3) (**b**) Tolerance to different concentrations of bile salt i.e., 0.1%, 0.5% and 1%. Each value represents the mean value ±standard deviation (SD) (*n* = 3). Bars with different lower-case letters are significantly different (*p* < 0.05).

Isolated strains have the ability to survive at pH 2 and pH 3 during 2 h of incubation (Figure 1a). At pH 2 the survival rate was lower as compared to pH 3 for all the tested isolates. The survival rate of B-2, B-5, B-11, B-19 and B-21 remained above 60% at pH 2 and pH 3 during 2 h of incubation.

Results also revealed that B-5, B-11 and B-19 showed significantly (*p* < 0.05) high resistance to gastrointestinal conditions (75%, 85% and 77%, respectively) at pH 2. All the isolates have the ability to tolerate various concentrations of bile salt (0.3%, 0.5%, and 1%) during 3 h of incubation (Figure 1b). Maximum survival was observed at 0.3% bile salt concentration while the least was observed at 1% bile salt concentration. As the concentration of bile salt increases the survival rate decreases. The strain B-11 survived well even such a high concentration of bile salt (1%) than any other strain. The potential of isolates to tolerate 0.4% phenol is shown in Table 2. All the isolates have the ability to tolerate 0.4% phenol after 24 h of incubation. Result revealed that non-significant difference was observed in the viable cell count after 24 h of incubation with minimum log reduction (<1 log CFU/mL).

#### 3.2.2. Production of Exopolysaccharides (EPS)

The potential of isolates to produce EPS was shown in Table 2. All the isolates were able to produce EPS but each strain exhibited different EPS production capacities. All the tested isolates had the ability to produce exopolysaccharides when grown on MRS agar supplemented with 2% (v/v) glucose as a carbon source. Among the isolates, B-11 produced the highest EPS (123 mg/L) followed by B-5 (112 mg/L) and B-21 (102 mg/L), while the least production was observed in B-19 (56 mg/L) and B-28 (61 mg/L).

#### 3.2.3. Auto-Aggregation Assay

**Figure 2:**
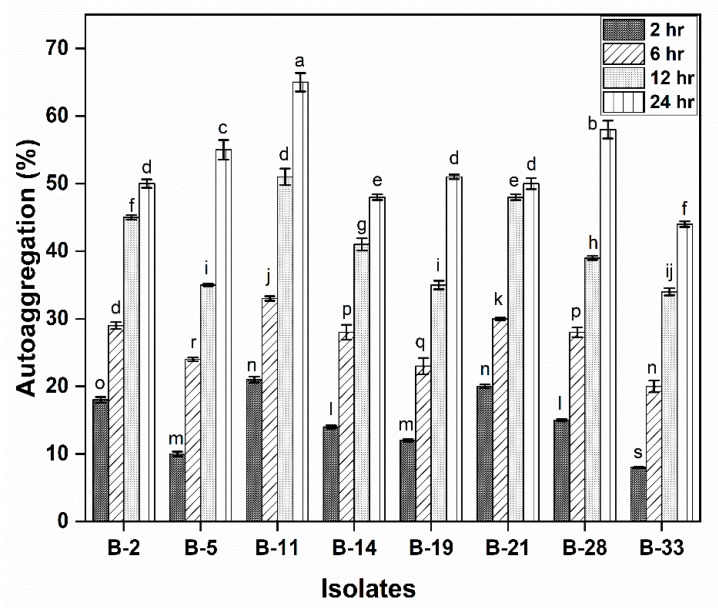
Autoaggregation (%) of isolates is calculated after 2 h, 6 h, 12 h and 24 h of incubation. Each value represents the mean value ± standard deviation (SD) (*n* = 3). Bars with different lower-case letters denoted significantly different (*p* < 0.05).

Cell surface traits (auto-aggregation and cell surface hydrophobicity) of probiotics are imperative for bacterial colonization and protection. All the tested isolates had high auto-aggregation activity. The isolate B-11, B-21 showed maximum auto-aggregation (65% and 60%) while the least was observed in B-33 (44%). However, auto-aggregation increases as the incubation time increases, so all the isolates showed an increase in auto-aggregation after 24 h of incubation (Figure 2). 

#### 3.2.4. Cell Surface Hydrophobicity 

The isolates B-11, B-5, B-28, B-19, and B-33 had high cell surface hydrophobicity (78.89%, 74.21%, 71.99%, 67.93%, and 60.77%) as compared to other isolates. However, isolate B-14 had the lowest hydrophobicity (45.21%) (Table 2).

#### 3.2.5. DPPH Free Radical Scavenging Activity 

isolate b-11 had the highest dpph scavenging ability (87.72%), followed by b-5 (84.44%), b-28 (81.19%), b-2 (80.29%), b-33 (80.72%), b-21 (79.74%) and b-19 (70.23%) (Table 2).

#### 3.2.6. Resistance to Hydrogen Peroxide

**Figure 3:**
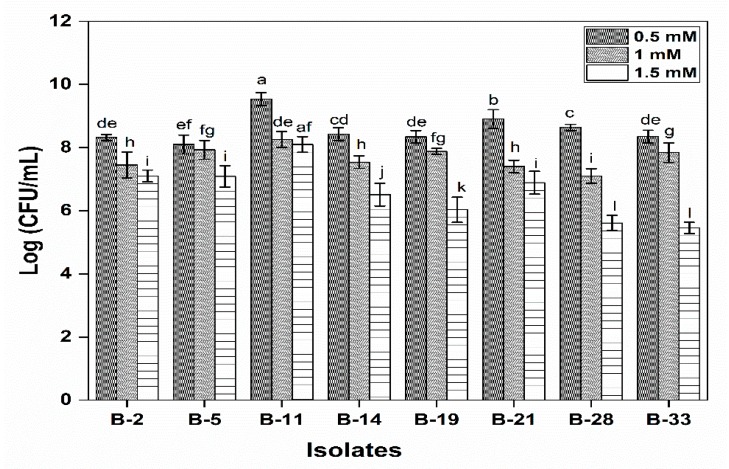
Resistance to hydrogen peroxide is determined by calculating the bacterial count of isolates at different concentration of hydrogen peroxide i.e., 0.5 mM, 1 mM and 1.5 mM. Each value represents the mean value ± standard deviation (SD) (*n* = 3). Bars with different lower-case letters denoted significantly different (*p* < 0.05).

All the tested isolates have the ability to resist H_2_O_2_. Results revealed that a significant impact on the viability of isolates was observed as the concentration of H_2_O_2_ increases exhibiting a decreasing trend. B-11 showed that the maximum viable cells count (8.1 log CFU/mL) while the least was observed in B-33 (5.45 log CFU/mL) at 1.5 mM H_2_O_2_ (Figure 3). In the case of B-2, B-5, B-14, B-19 and B-21 the viable cell count was >10^6^ log CFU/mL.

#### 3.2.7. Depletion of Sodium Nitrite

Nitrite is used as an additive in a number of fermented products. It is still considered undesirable in food products due to its safety concerns. It is recognized as a carcinogenic and teratogenic substance due to its high oxidative and reductive activities. There is a need to eliminate nitrite during fermentation. The results propounded in Table 2 revealed that all isolates had the ability to deplete nitrite. Among the isolates, B-11 depleted maximum nitrite 84% followed by B-5 (76%) while the least nitrite depletion was observed in B-21 (50%).

#### 3.2.8. Antibacterial Activity

The results of the antibacterial activity as a halo of growth inhibition produced on agar plates by tested isolates against indicator pathogens were presented in Table 3. Isolates exhibited strong antimicrobial activity against pathogenic bacteria (*Escherichia coli*, *Salmonella typhimurium* and *Staphylococcus aureus*). Most of the isolates inhibited indicator pathogenic bacteria and the zone of inhibition was >6 mm. The isolates B-19 and B-28 exhibited zero inhibition against *Escheria coli* and *Salmonella typhimurium*, respectively. The observed inhibitory property of the isolates could be attributed due to the production of antimicrobial compounds. Based on the results, *E. coli* and *Salmonella typhimurium* were the most sensitive and *S. aureus* was the most resistant indicator bacteria against the tested isolates.

#### 3.2.9. Cholesterol Reduction Assay

**Figure 4:**
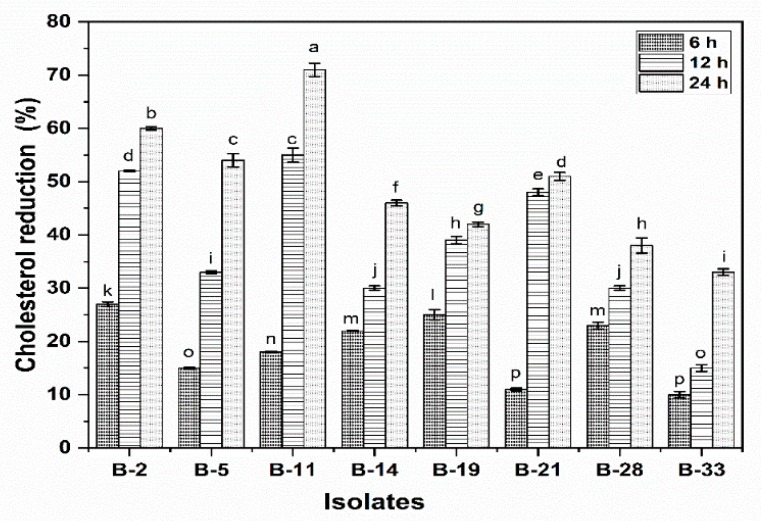
Cholesterol reduction (%) of isolates after 6 h, 12 h and 24 h of incubation. Each value represents the mean value ± standard deviation (SD) (*n* = 3). Bars with different lower-case letters denoted significantly different (*p* < 0.05).

The removal of cholesterol by 8 probiotics bacterial strains grown on MRS broth supplemented with 0.3% oxgal was presented in Figure 4. All the tested isolates were able to assimilate cholesterol. Results revealed that cholesterol removal varied significantly (*p* < 0.05) among all the isolates at the same incubation time i.e., 6 h, 12 h and 24 h. Probiotic strains B-2, B-5, B-11 and B-21 showed significantly (*p* < 0.05) high cholesterol removal (60%, 54%, 71% and 51%, respectively) after 24 h of incubation as compare to other isolates. The lowest cholesterol removal ability was observed in isolate B-28 (38%) and B-33 (33%). The cholesterol removal ability increases as the incubation time increases.

### 3.3. Safety Evaluation of Isolates

#### 3.3.1. Antibiotics Susceptibility 

The antibiotic susceptibility of the tested isolates was documented by using different commonly used antibiotics. Results presented in Table 4 revealed that all the isolates were resistant to nalidixic acid, fusidic acid, polymyxin B, neomycin, streptomycin, gentamicin, rifampicin and kanamycin except B-14 which exhibited sensitivity to neomycin, B-21 was sensitive to rifampicin and streptomycin, respectively.

#### 3.3.2. Hemolytic Activity 

Hemolytic activities of 8 tested isolated were evaluated on blood agar plates. None of the tested strains showed α-hemolytic and ß-hemolytic activity when grown on Columbia blood agar plates. The tested strains showed γ hemolytic, i.e., negative, or no hemolytic activity.

#### 3.3.3. Cytotoxicity

**Figure 5:**
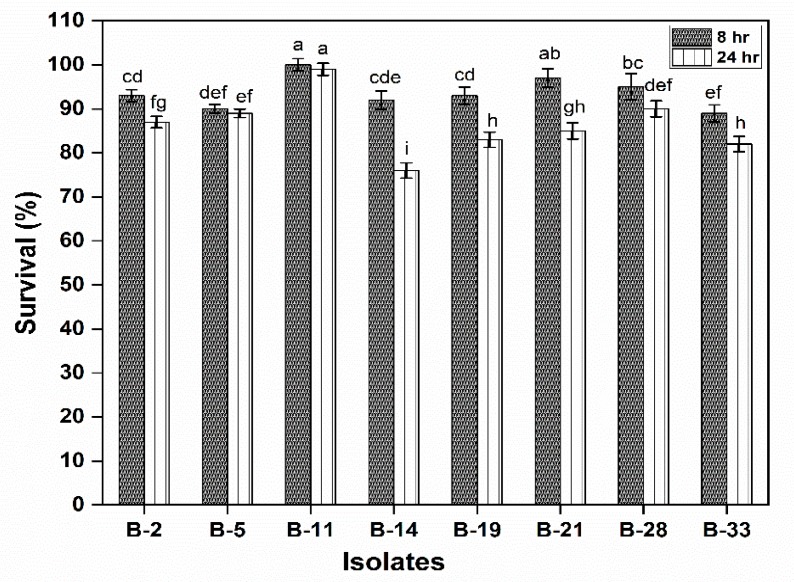
Cytotoxicity to observed the survival (%) of isolates after 8 h and 24 h of incubation. Each value represents the mean value ± standard deviation (SD) (*n* = 3). Bars with different lower-case letters denoted significantly different (*p* < 0.05).

In this experiment, MTT [3-(4,5-Dimethylthiazol-2-yl)-2,5-diphenyltetrazolium bromide] assay was used to observe the effect of different isolates on Caco-2 cell viability. The results showed that all the tested isolates did not significantly damage cells after 8 h of incubation however, 24 h of incubation harmed the cells and cells lost their viability. There was no significant difference in cell viability of B-11 after 24 h of incubation (Figure 5). Furthermore, least damage was observed in the case of B-14, B-19 and B-21 after 24 h of incubation. Therefore, the probiotic treatment showed insignificant changes among all the tested isolates in the culture supernatant of Caco-2 cells, keeping the membrane integrity during probiotic exposure after 8 h but the viability decreases significantly between 8 to 24 h. 

### 3.4. Molecular Identification of Isolate (B-11)

Considering that among all the isolates, B-11 possessed most of the probiotic’s attributes. The isolate was further confirmed through 16S rRNA gene sequence analysis. On the basis of molecular identification, purified PCR products were sequenced and compared with the information in the NCBI database. BLAST search disclosed that isolated bacterium as *B. longum* subsp. *longum* which exhibited a similarity index (100%) to other *B. longum* strains. Therefore, the isolate was a strain of *B. longum* and designated as *B. longum* B-11. The 16S rRNA sequence was deposited to NCBI Genebank data base under the accession number MH041649.1.

### 3.5. Scanning Electron Microscopy of Rat Intestine

**Figure 6:**
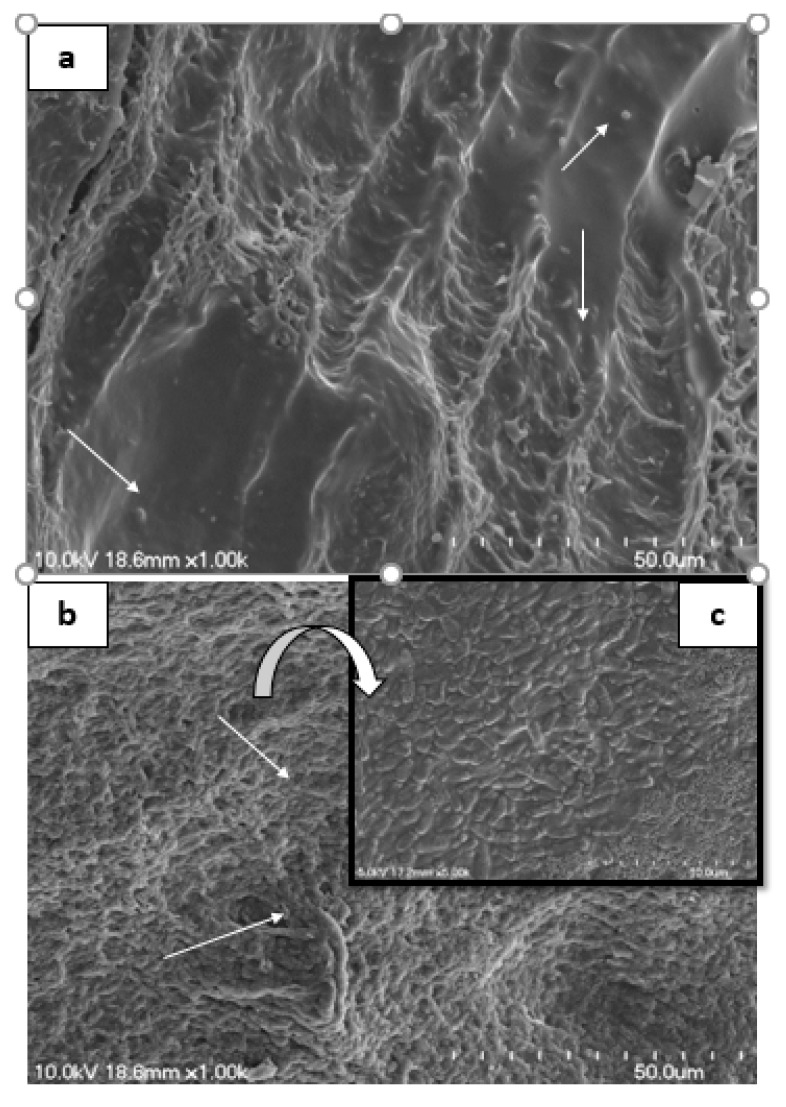
Scanning electron microscopy (SEM) of excised rat GIT showing presence of large number of coccus-shaped bacteria on the upper surface (G_0_) (**a**). The rat administrated with *B. longum* B-11 displayed huge number of rods on the microvilli of GIT (G_1_) (**b**). Magnified images of *B. longum* B-11 showing bacterial cell are V or Y shaped rods (**c**).

Scanning electron microscopy images revealed that small intestine of rat (control group, G_0_) showed a large number of coccus shaped bacterial attachment on the upper surface (Figure 6a), while the rats treated with *B. longum* B-11 (G_1_) showed presence of a large number of rods shaped bacteria (*B. longum* B-11). Well organized and steady microvilli can be observed in the case of *B. longum* B-11 treated rats (Figure 6b,c).

## 4. Discussion

In the present study, 35 colonies were isolated from camel milk and eight (B-2, B-5, B-11, B-14, B-19, B-21, B-28, B-33) was identified as bifidobacteria on the basis of morphology, physiology and biochemical characterization. Studies revealed that *Bifidobacterium* was Gram-positive, rods (V or Y) shaped, catalase-negative, non-motile and non-spore forming [4]. 

The probiotic potential of isolates was assessed through their survival ability under the artificial simulated conditions in the digestive tract. Various researchers contributed their work in this field to evaluate potentially probiotic strains. pH is an important parameter that affects the growth and viability of probiotics during gastric transit. Among eight isolates, three isolates (B-2, B-11, B-19) showed survival rate in the range of (75–85%) at pH 2 and more than 90% at pH 3 after 2 h of exposure. The survival rate is higher as compared to previously reported *Bifidobacterium* strains under such low pH [42,43]. 

Probiotics should survive in the gastrointestinal conditions and reach alive in the small intestine, where they colonize and impart positive health benefits to the host. The decreased in the viability and survival is due to the presence of pepsin enzymes and low pH of gastric juice [44]. The probiotics should with stand harsh gastrointestinal conditions (gastric juice and bile salt) to exert positive health benefits to humans. Gastric juice and bile salt-resistance is considering one of the prime criteria in the selection of probiotics [45]. *B. longum* strains presented maximum viability in GIT as compared to other species belonging to the same genera [42].

All the isolates exhibited more than 70% survival rate at 0.1% (w/v) bile salt concentration except B-19. Three isolates (B-2, B-11, B-28) showed a survival rate in the range of (70–75%) at such a high concentration of bile salt (1%). Sanchez, et al. [46] studied the probiotic potential of different *B. longum* strains (mutant and wild type) at different bile salt concentrations (0.5%, 1%, 2% and 3%) and expounded that viability of *B. longum* decreased as bile salt concentration increased. Mutant strains indicated viability at 0.5% to 3% bile salt concentration while the wild type was not able to grow at such a high concentration of bile salt. Haros, et al. [47] investigated tolerance of *B. pseudocatenulatum* to (0.5–2%) bile salt and reported that *Bifidobacterium* strain was resistant to bile salt even after 4 h of incubation. Ren, Li, Qin, Yin, Du, Ye, Liu, Liu, Wang, Li, Sun, Li, Tian and Jin [10] demarcated similar results for bile salt tolerance at 0.3% to 0.5% concentration. Even most of the probiotic strains showed resistance to 1% bile salt, which is approximately three times the bile concentration in the human intestine. Haghshenas, et al. [48] observed tolerance to bile salt but survival decreased as a function of incubation time. In the current study, all isolates have the ability to tolerate high phenol concentration (0.4%), revealed that they can tolerate the bacteriostatic effect of phenol in GIT. Phenol is actually the by-product of metabolism of aromatic amino acids that produce in the gut. 

Exopolysaccharide production is important for protection, colonization and acts as intermediaries to establish an association between bacteria and host [49]. All the isolates were able to produce EPS. Among *Bifidobacterium* strains, *B. longum* is the most frequently cited probiotics due to the comparatively high yield of EPS being produced. Prasanna, et al. [50] screened various *Bifidobacterium* strains and reported that among all the *Bifidobacterium* strains, *B. longum* subsp. *infantis* CCUG 52,486 was the highest EPS producing strain (138 mg/L). The capsular structure of EPS protects probiotics from harsh gastrointestinal conditions like gastric juice, bile salt and etc. High yielding EPS strains could survive well in high acidic and bile salt conditions. In this way, EPS promotes bacterial growth and survival in the presence of high acid and bile salt conditions [51]. EPS also serves as a growth substrate for gut microflora and provides protection against phagocytosis and bacteriophage attack. 

Cell surface properties i.e., cell surface hydrophobicity and autoaggregation are important for bacterial attachment to intestinal lining and colonization in the GIT. In the current study, B-5 (74.21%), B-11 (78.89%) and B-28 (71.99%) exhibited comparatively high hydrophobicity to xylene. These isolates also showed high autoaggregation B-5 (55%) B-11 (65%) and B-28 (58%) after 24 h of incubation. Xu, Jeong, Lee and Ahn [28] described cell surface property and reported that the highest value for hydrophobicity was found in *B. longum* B6 (53.6%) followed by *Lb. rhamnosus* GG (46.5%). Moreover, Rahman, et al. [52], also explored 13 strains of 4 different *Bifidobacterium* spp. for their auto-aggregation ability and surface hydrophobicity. They explained that among all *Bifidobacterium* spp. *B. longum* presented high cell surface hydrophobicity. Auto-aggregation ability and surface hydrophobicity are related to cell adhesion, which promotes binding of probiotics to the intestinal lining. As a result of this binding, it acts as a barrier and pathogens are unable to colonize. This adhesion of probiotics to the intestinal cell is essential for pathogen omission and immunomodulation [53]. In vitro, cell surface properties are not a reliable approach to judge their interaction within the host cell. There is still a need to study it in vivo, for a better understanding of this interaction. 

*Bifidobacterium* has the ability to act as an antioxidant by quenching free oxygen radicles. In the current study the cell-free supernatant of B-2 (80.29%), B-5 (84.44%), B-11 (87.72%), B-28 (81.19%), B-33 (80.72%) exhibited strong antioxidant activity as compared to other studies. Shen, et al. [54] documented the antioxidant activity of *B. animals* 01 both in vitro and in vivo. The results reported that culture supernatant exhibited the highest DPPH radical scavenging activity (73.11%). Likewise, Wang, et al. [55] elucidated that, LAB and *Bifidobacterium* fermented soymilk had higher antioxidative activity than un-fermented soymilk. Nevertheless, these studies represented the antioxidative ability in vitro but there is still a need to study this activity in vivo. Synthetic antioxidants have serious health implications regarding their safety and long term utilization. Synthetic antioxidants i.e., butylated hydroxytoluene (BHT) and butylated hydroxyanisole (BHA) have toxic and carcinogenic effects [56]. Therefore, it is the need of the day to explore non-toxic, natural and low-cost antioxidants as a substitute for a synthetic antioxidants in pharmaceutical and food industries. 

Nitrite is commonly used as a food additive and has been widely found in various food products. It is an important N-nitrosamines precursor, which potentially causes cancer and methemoglobinemia. Nitrites also react with amines (protein degradation products) to form N-nitroso compounds [10]. So, it is an important safety concern to limit the use of nitrite in food products. In this context, all the tested isolates have the ability to deplete nitrite and restrict the conversion of nitrite into nitrosamines. Although the exact mechanism is still not fully understood it might be due to enzymatic and chemical reactions. There is a need to further evaluate this in a food product. 

Probiotics are helpful in the management of GIT infections. They exert positive effects against antibiotic-associated diarrhea, travel diarrhea and rotavirus. These antimicrobial substances are proteinases in nature and produced by various *Bifidobacterium* spp. Collado, et al. [57] explicated that antimicrobial peptides produced from *Bifidobacterium* strains had strong antagonist activity against both antibiotic sensitive and antibiotic-resistant pathogens. Antimicrobial peptides could be one of the effective mechanisms to combat infectious diseases. 

An isolate B-11 exhibited most of the probiotics attributes and confirm through molecular identification and named as *Bifidobacterium longum* B-11. The obtained sequence was deposited into NCBI GenBank and allotted NCBI accession number (MH041649.1). *B. longum* B-11 was further selected for in vivo attachment to the intestinal lining and the results are in line with the findings of Choudhary, Dubey, Sengar and Dheeman [40]. *B. longum* B-11 was the potential microorganism to be used as probiotics for human consumption due to better colonization in GIT and better adhesion to the epithelial lining of the host. 

High cholesterol level has several health implications i.e., high risk of cardiovascular ailments which is the major cause of death. Use of the drug in the treatment of hypercholesterolemia has numerous side effects. Therefore, it is of utmost important to use natural, cost-effective methods to reduce serum cholesterol. Consumers are more concerned to use safe and alternate products for hypercholesterolemia that limit dependence on drug therapy. In this context, the use of supplementing diets with probiotic strains is one of the effective and promising strategies to reduce serum cholesterol. The results of the current study revealed that *Bifidobacterium* strains have the ability to lower down the cholesterol level. *B. longum* B-11 exhibited maximum cholesterol removal percentage. The result of the current study is in accordance with the findings of El-Gawad, et al. [58], who evaluated that the cholesterol reduction of *B. longum* Bb-46 fortified buffalo milk yogurts. They fed hypercholesterolemic male albino rats with 50 g yoghurt containing 0.07% (w/v) probiotics for 35 consecutive days. They delineated that probiotic fortified yoghurt significantly reduced total cholesterol level by 50.3%, triglycerides by 51.2% and LDL by 56.3% as compared to control. Likewise, Xiao, et al. [59] explicated the effect of low fat yoghurt fortified with *B. longum* BL1 (10^8^ CFU/g) on cholesterol level of thirty-two subjects (cholesterol level 220–280 mg/dL, aged 28–60 years old, weight 55.4–81.8 kg). The results illustrated that there was a significant reduction in serum total cholesterol level, LDL cholesterol and triglycerides as compared to control after 4 weeks. 

As far as safety is concerned the isolates should have the ability to resist commonly used antibiotics [10]. The literature revealed that *Bifidobacterium* is susceptible to antibiotics and can be consumed safely after antibiotic therapy for maintaining the balance of gut microflora. Similarly, Zhou, et al. [60] investigated that *B. lactis* strains were susceptible to erythromycin, novobiocin, rifampicin, tetracycline, spectinomycin, h-lactam, chloramphenicol, penicillin, cephalothin and ampicillin. These strains were resistant towards nalidixic acid, fusidic acid, polymyxin B, aminoglycosides, neomycin, streptomycin, gentamicin and kanamycin. Likewise, Temmerman, et al. [61] isolated *B. longum* from different products and evaluated that *B. longum* was resistant to kanamycin and susceptible to erythromycin, tetracycline, chloramphenicol, penicillin and vancomycin.

According to the European Food Safety Authority (EFSA), the evaluation of hemolytic activity is strongly recommended if the isolated bacteria intended to use in food products, even if they have GRAS or QPS (Quality Presumption of Safety) status (FAO/WHO 2006). In this study, the hemolytic activity of 8 tested isolates was evaluated on Columbia blood agar plates. None of the tested strains showed α- hemolytic and β-hemolytic activity when grown in Columbian sheep blood agar. All the tested strains showed γ- hemolytic or no hemolytic activity. Our results were in agreement with the finding of Oh and Jung [62] who determined the hemolytic activity of five *Lactobacillus* species isolated from traditionally fermented millet-based alcoholic beverages. The results of current findings were also consistent with the results of Wang, et al. [63] who evaluated the probiotic potential of lactic acid bacteria from Chinese spontaneously fermented non-dairy food products and revealed no hemolytic activity of probiotic. Many researchers [37,64,65,66] reported that probiotics did not showed hemolytic activity. When the safety of probiotic is concerned, lack of hemolytic activity is important during the selection of probiotic strains, because such strains are non-virulence and lack of hemolysin ensures that virulence will not appear among the bacterial strains (FAO/WHO 2006). The enzymes production that are capable of degrading mucin is suggested as a key factor of virulence for pathogens. So that, this feature is not recommended for probiotic strains because it modifies the intestinal mucosal lining as a result mucosal invasion happens by other toxic materials and pathogens [37].

It is proved that all the tested isolates were found to have better anticancer activity with cell viability > 80% of the Caco-2 cell. Thus, the novelty of isolates especially B-11 has been proved for its effective anti-carcinogenic effect. Chui and coworkers reported similar results, *L. plantarum* co-cultured with Caco-2 cells did not affect cell viability within 10 h, but cell viability decreased significantly between 12 and 14 h but the decreases was non-significant [67]. The inhibitory effects of probiotics upon the proliferation of several colon cancer cell lines have been previously demonstrated by various authors [38,68,69,70,71,72,73].

MTT assay is considered as a highly sensitive assay for determining cellular respiration, cell viability and cytotoxicity because only living cells are able to produce formazan products during this reaction. Lactobacilli and bifidobacteria are the most prominent probiotics that have been recognized as safe and increasing research interest due to their significant role in the prevention of cancer [74]. Probiotics generally modulate intestinal microflora, inactivate carcinogenic complexes, improves host’s immunity, compete with pathogens, have anti-proliferative effects by regulating apoptosis and cell differentiation [68].

## 5. Conclusions

In the current study, eight potential probiotics were successfully isolated from raw camel milk according to the international probiotic guidelines. Among all the isolates, B-11 had the maximum potential for practical application due to high resistance to GIT conditions as well as strong antagonistic and antioxidant activities. B-11 also displayed antibiotic susceptibility and produced the highest EPS yield. B-11 is effective to deplete nitrite and assimilate cholesterol. Therefore, B-11 is introduced as a potential probiotic to be used safely in the development of functional food products. Moreover, in vivo studies would be needed to further evaluate its immunomodulatory, safety and health perspective as a potential probiotic.

## Figures and Tables

**Table 1:** Morphological, physiological and biochemical characterization of the isolates.

Tests	Isolates							
	B-2	B-5	B-11	B-14	B-19	B-21	B-28	B-33
Morphology	V shaped rod	rod	Y- shaped rod	V-shaped rod	rod	rod	V-shaped rod	rod
Gram Staining	+	+	+	+	+	+	+	+
Catalase test	-	-	-	-	-	-	-	-
Growth at different temperature								
10 °C	-	+	+	+	-	+	-	+
37 °C	+	+	+	+	+	+	+	+
45 °C	+	-	+	+	+	+	-	+
Growth at different NaCl concentration								
3%	+	+	+	+	+	+	+	+
5%	+	-	+	+	-	+	-	+
7%	+	-	-	-	-	+	-	-
Growth at different pH								
4.4	+	+	+	+	+	+	+	+
9.6	-	-	-	-	-	+	-	-
Motility test	-	-	-	-	-	-	-	-
Endospore test	-	-	-	-	-	-	-	-
CO_2_ from glucose	-	-	-	-	-	-	-	-
Carbohydrate fermentation								
Glucose	+	+	+	+	+	+	+	+
Maltose	-	+	+	-	+	+	+	+
Lactose	+	+	+	+	+	-	-	+
Galactose	+	+	-	-	-	+	+	+
Raffinose	+	+	+	+	+	+	+	+
Sorbitol	-	+	-	-	+	+	+	+
Sucrose	+	-	+	+	-	-	-	+
Xylose	+	+	+	-	-	+	+	+
Fructose	+	+	+	-	+	+	+	+

**Table 2:** Phenol tolerance, EPS production, Cell surface hydrophobicity, DPPH free radical scavenging activity and Depletion of sodium nitrite by the isolates (means ± SD).

Isolates	Phenol Tolerance (log CFU/mL)		EPS Production (mg/L)	Cell Surface Hydrophobicity (%)	DPPH Free Radical Scavenging Activity (%)	Depletion of Sodium Nitrite (%)
	0 h	24 h				
B-2	8.65 ± 0.14	8.32 ± 0.14	91 ± 2.41	56.89 ± 1.59	80.29 ± 3.12	65 ±0.08
B-5	8.32 ± 0.43	8.21 ± 0.23	112 ± 2.53	74.21 ± 3.12	84.44 ± 2.93	76 ± 0.14
B-11	8.54 ± 0.56	8.45 ± 0.41	123 ± 3.92	78.89 ± 2.43	87.72 ± 3.01	84 ± 0.13
B-14	8.92 ± 0.80	8.12 ± 0.17	80 ± 2.02	45.21 ± 0.91	41.38 ± 0.42	54 ± 0.19
B-19	8.46 ± 0.21	7.94 ± 0.21	56 ± 1.34	67.93 ± 1.25	70.23 ± 1.40	74 ± 0.13
B-21	8.53 ± 0.11	8.39 ± 0.19	102 ± 2.10	49.34 ± 2.51	79.74 ± 2.52	50 ± 0.09
B-28	7.91 ± 0.14	7.59 ± 0.10	61 ± 3.21	71.99 ± 2.13	81.19 ± 2.16	79 ± 0.31
B-33	8.12 ± 0.10	7.98 ± 0.34	75 ± 2.37	60.77 ± 1.76	80.72 ± 2.34	69 ± 0.56

**Table 3:** Antibacterial activity of isolates (means ± SD).

	Zone of Inhibition		
Isolates	*Staphylococcus aureus* (mm)	*Salmonella typhimurium* (mm)	*Escherichia coli* (mm)
B-2	8 ± 0.51	8 ± 0.11	7 ± 0.26
B-5	10 ±0.42	7 ± 0.09	10 ± 0.21
B-11	11 ± 0.35	12 ±0.12	14 ± 0.13
B-14	8 ± 0.14	10 ± 0.21	11 ± 0.12
B-19	8 ± 0.21	9 ± 0.08	0 ± 0.27
B-21	7 ± 0.28	10 ± 0.12	6 ± 0.06
B-28	9 ± 0.17	0 ± 0.10	8 ± 0.15
B-33	6 ± 0.06	6 ± 0.04	9 ± 0.19

Where, no inhibition diameter equal to 0 mm; weak inhibition, diameter between 0–3 mm; good inhibition, diameter between 3–6 mm; strong inhibition, greater than 6 mm.

**Table 4:** Antibiotic susceptibility of isolates.

Antibiotics	Disc Potency (µg)	B-2	B-5	B-11	B-14	B-19	B-21	B-28	B-33
Ampicillin	10	S	S	S	S	S	S	S	S
Cephalothin	15	S	S	S	S	S	S	S	S
Chloramphenicol	50	S	S	S	S	S	S	S	S
Cloxacillin	20	S	S	S	S	S	S	S	S
Erythromycin	10	S	S	S	S	S	S	S	S
Fusidic acid	10	R	R	R	R	R	R	R	R
Gentamicin	10	R	R	R	R	R	R	R	R
Kanamycin	30	R	R	R	R	R	R	R	R
Nalidixic acid	20	R	R	R	R	R	R	R	R
Neomycin	20	R	R	R	S	R	R	R	R
Novobiocin	30	S	S	S	S	S	S	S	S
Penicillin	10	S	S	S	S	S	S	S	S
Polymyxin B	20	R	R	R	R	R	R	R	R
Rifampicin	20	R	R	R	R	R	S	R	R
Spectinomycin	10	S	S	S	S	S	S	S	S
Streptomycin	25	R	R	R	R	R	S	R	R
Tetracycline	30	S	S	S	S	S	S	S	S
Vancomycin	30	S	S	S	S	S	S	S	S

Where, inhibition zone diameter <8 mm, resistant (R); inhibition zone diameter between 8–10 mm, moderate (M) and inhibition zone diameter >10 mm, susceptibility (S).
